# Note on the Induction of Sleep and Anæsthesia by Compression of the Carotids

**Published:** 1855-06

**Authors:** Alexander Fleming

**Affiliations:** Professor of Materia Medica, Queen’s College, Cork


Note on the Induction of Sleep and Anæsthesia by Compression of the Carotids. By Alexander Fleming, M. D., Professor of Materia Medica, Queen's College, Cork.—While preparing a lecture on the mode of operation of narcotic medicines, I thought of trying the effect of compressing the carotid arteries on the functions of the brain. I requested a friend to make the first experiment on my own person. He compressed the vessels at the upper part of the neck, with the effect of causing immediately deep sleep. This experiment has been frequently repeated on myself with success, and I have made several cautious but successful trials on others. It is sometimes difficult to catch the vessels accurately but once fairly under the finger, the effect is immediate and decided.

There is felt a soft humming in the ears, a sense of tingling steals over the body, and, in a few seconds, complete unconsciousness and insensibility supervene, and continue so long as the pressure is maintained. On its removal, there is confusion of thought, with return of the tingling sensation, and in a few seconds consciousness is restored. The operation pales the face slightly, but the pulse is little, if at all affected. In profound sleep the breathing is stertorous, but otherwise free. The inspirations are deeper. The mind dreams with much activity, and a few seconds appear as hours, from the number and rapid succession of thoughts passing through the brain. The experiments have never caused nausea, sickness, or other unpleasant symptom, except, in two or three instances, languor. The period of profound sleep, in my experiments, has seldom exceeded fifteen seconds, and never half a minute.

The best mode of operating is to place the thumb of each hand under the angle of the lower jaw, and feeling the artery, to press backwards, and obstruct the circulation through it. The recumbent position is best, and the head of the patient should lie a little forwards, to relax the skin. There should be no pressure on the windpipe.

The internal jugular vein must be more or less compressed at the same time with the carotid artery; and it may be thought that the phenomenon is due, wholly or in part, to the obstructed return of blood from the head. I am satisfied that the compression of the artery, and not of the vein, is the cause. The effect is most decided and rapid when the arterial pulsation is distinctly controlled by the finger, and the face loses somewhat of its color; and, on the other hand, is manifestly postponed and rendered imperfect when the compression causes congestion of the countenance.

This mode of inducing anæsthesia is quick and certain. The effects diminish immediately when the arteries are relieved from pressure, and are not liable to increase, as happens sometimes with chloroform and ether, after the patient has ceased to respire the vapors. So far as my experience goes, it has shown no tendency to cause faintness; and usually, after its employment, no vnpleasant feeling whatever remains.

I think it may be found useful as a remedial agent in certain headaches, tetanus, asthma, and other spasmodic diseases, and to prevent pain in such small operations as the extraction of a tooth or the opening of an abscess. Whether the compression can be continued *with safety* sufficiently long to make it available in larger operations, has to be ascertained. But, whatever be the practical value of this observation, it is at least interesting as a physiological fact, and may be the means of throwing light on the causes of ordinary, medicinal, and hypnotic sleep, and of coma. Some facts encourage the supposition that the circulation of the brain is languid in ordinary slumber, and the etymology of the word carotid shows the ancient belief in the dependence of deep sleep on some interference with the passage of the blood througe these vessels; and it is not an unreasonable conjecture, that hypnotic sleep may be sometimes caused or promoted by the contracted muscles and constrained position of the neck compressing the carotid arteries, and diminishing the supply of blood to, and pressure on, the brain.—*Medico-Chirurgical Review.*

